# All-into-one strategy to synthesize mesoporous hybrid silicate microspheres from naturally rich red palygorskite clay as high-efficient adsorbents

**DOI:** 10.1038/srep39599

**Published:** 2016-12-21

**Authors:** Wenbo Wang, Guangyan Tian, Dandan Wang, Zhifang Zhang, Yuru Kang, Li Zong, Aiqin Wang

**Affiliations:** 1 Key Laboratory of Clay Mineral Applied Research of Gansu Province, Center of Eco-materials and Green Chemistry, Lanzhou Institute of Chemical Physics, Chinese Academy of Sciences, Lanzhou 730000, P.R. China; 2 R&D Center of Xuyi Palygorskite Applied Technology, Lanzhou Institute of Chemical Physics, Chinese Academy of Sciences, Xuyi 211700, P.R. China; 3 University of the Chinese Academy of Sciences, Beijing 100049, P.R. China

## Abstract

A mesoporous hybrid silicate microsphere with superior adsorption performance has been successfully synthesized by employing an “all-into-one” strategy and a simple one-pot hydrothermal process using naturally abundant low-grade red palygorskite (PAL) clay as raw material in the presence of non-toxic SiO_3_^2−^ and Mg^2+^ ions. As is expected, both the PAL and associated minerals transformed into a new amorphous mesoporous hybrid silicate microsphere without using any additional pore-forming template. The mesoporous silicate microsphere shows a large pore size of 37.74 nm, high specific surface area of 489.81 m^2^/g (only 54.67 m^2^/g for raw PAL) and negative surface potential of −43.3 mV, and its maximum adsorption capabilities for Methylene bule (MB) and Crystal violet (CV) reach 407.95 mg/g and 397.22 mg/g, respectively. Meanwhile, 99.8% of MB (only 53% for raw PAL) and 99.7% of CV (only 43% for raw PAL) were sucessfully removed from 200 mg/L of initial dye solution by only using 1 g/L of the adsorbent. In addition, the spent adsorbent can be easily regenerated and repeatly reused for muptiple cycles. The study on adsorption mechanism revealed that electrostatic attraction, hydrogen bonding and chemical complexing interactions are the main factors contributed to the high dye adsorption.

In greening the 21^st^ century materials world^1^, naturally available silicates have received unprecedented attention owing to their abundant, low-cost, stable, safe and environmentally benign advantages over most of the artificial materials^2^,^3^. The unique pore structure, ion-exchange capability, surface charge as well as plenty of functional groups of silicates make them able to capture various matters^4^,^5^,^6^,^7^. Therefore, silicates have shown great prospects to be used as cheap, high-efficient and safe adsorbents.

Palygorskite (PAL), a special member in natural silicate families, has drawn much attention in both of the academia and industry as an efficient adsorbent due to its unique crystal structure. PAL is a hydrated magnesium-rich silicate clay mineral with nanorod-like crystal morphology and nano-channels (cross-sectional area is 0.37 nm × 0.64 nm) along the *c*-axis of rod^8^,^9^. It has a 2:1 ribbon-layer structure composed of two continuous tetrahedron sheets and one discontinuous octahedron sheet, in which the ribbon is connected to each other by the inversion of SiO_4_ tetrahedron along a set of Si-O-Si bonds (Fig. S1, Supplementary Information)^10^,^11^. The octahedral cations in the PAL crystal can be partially replaced by di- or tri-valent cations owing to isomorphic substitution phenomenon, which make PAL possessing permanent negative charges and exchangeable cations^12^. Owing to the special nanorod-like crystal morphology, excellent stability and rich surface groups, PAL has been widely applied in many aspects, such as nanocomposites^13^,^14^,^15^, optical material^16^, thermal insulation material^17^, and carriers of catalyst and activated carbon^18^,^19^,^20^. Besides, PAL has also been used as non-toxic, low cost and eco-friendly adsorbents for removal of dyes^21^, heavy metal ions^22^,^23^, and color matters^24^. Among them, the most promising application of PAL is believed to develop economic and efficient adsorbent. Unfortunately, most of the natural PAL minerals were formed from lacustrine deposit, which result in poor crystallinity of the PAL rod as well as generation of many associated minerals (i.e., clinochlore, quartz, dolomite, calcite)^25^. Although these PAL clay minerals have huge reserves and wide distribution in nature, their overall grade are very low. These low grade PAL minerals are not effectively utilized in industry, and even abandoned as solid wastes or tailings.

Major efforts have been made to improve the adsorption properties of natural PAL clays. Different physical and chemical methods, e.g., grinding, acid activation, heat activation, organification or surface grafting, have been employed to modify PAL to improve its adsorption performance. For example, Liu *et al*. treated PAL clay with grinding for efficient adsorption of methylene blue^26^. Chen *et al*. modified PAL by a heat treatment process to improve its adsorption capacity for dyes^27^. Frini-Srasra and Srasraa reported by using acid activation method to improve the adsorption capability of PAL to Cd(II)^28^. Zhou *et al*. modified PAL with polyamidoamine dendrimer to improve its chelating capacity to both the Pb(II) and dye^29^. It is well known that the primary factors limited the adsorption performance of low-grade PAL clay are: (i) the associated minerals in PAL clays show no adsorption activity; (ii) the low specific surface area (<100 m^2^/g) as well as negative charge of raw PAL; (iii) the scanty of Si(M)-OH groups, which is believed to response to the high adsorption capacity. Although the modification methods mentioned above are useful to improve the adsorption, still hard to reach a high level of adsorption capacity, since these conventional methods fail to improve the pore structure, surface charge, or surface area, and transform the associated minerals (i.e., quartz, kaolinite) as silicate with adsorption activity.

Our previous works confirmed that hydrothermal process is a more effective approach to tune the microscopic structure of PAL and enhance its adsorption properties than conventional surface modification^30^,^31^. By using the simple hydrothermal reaction, the inert Si-O-Si bonds in clay mineral will be broken to form Si-O^−^ groups with stronger holding capability to dyes or heavy metal ions. Other properties like pore structure and specific surface area will also be improved. Especially in recent years, great concerns have been paid on the hybrid materials with nano/micro structure^32^,^33^ and the mesoporous materials with excellent performance^34^,^35^,^36^. Hydrothermal process has been used to synthesize various metal silicate hybrid materials with different morphologies, porous structure and superior adsorption performance^37^,^38^,^39^. It has been demonstrated that the new metal silicates could be synthesized by a hydrothermal process using PAL clays as Si, Mg and Al sources without considering their grade. Therefore, fusing all the elements into one silicate (all-into-one) by tuning the ratio of elements through a one-pot hydrothermal process is feasible to produce new porous hybrid silicate adsorbent with high specific surface area and superior adsorption capacity. However, rare research concerns on the synthesis of a mesoporous silicate adsorbent from naturally abundant PAL clay.

As part of our systematic works about the development of high-efficient silicate adsorbent based on clay mineral, in this paper, we successfully synthesized mesoporous silicate microsphere adsorbent using low-grade PAL clay as raw materials under hydrothermal condition by an “all-into-one” strategy. The structure of the adsorbents was studied by Fourier transform Infrared spectra (FTIR), X-ray diffraction (XRD), Field emission scanning electron microscope (SEM) and BET analysis, and the adsorption properties of the mesoporous adsorbent were evaluated using Methylene blue (MB) and Crystal violet (CV) as the model dyes.

## Results and Discussion

### SEM and TEM morphologies

**Figure 1:**
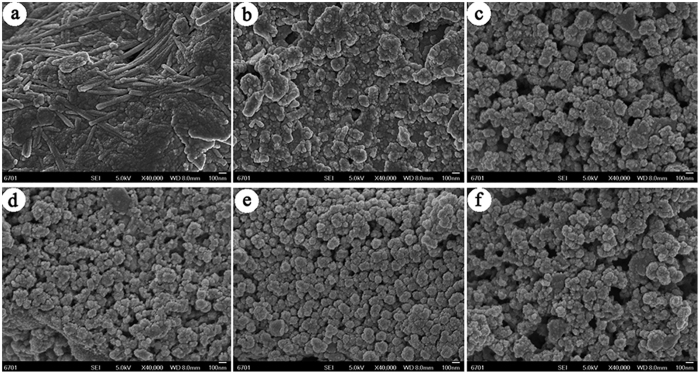
The SEM images of (**a**) RPAL and the adsorbents (**b**) SiMg-21-2, (**c**) SiMg-21-4, (**d**) SiMg-21-8, (**e**) SiMg-21-12, and (**f**) SiMg-21-24.

Figure 1 shows the SEM images of RPAL and the hybrid silicate adsorbents prepared at different reaction times. As shown in Fig. 1a, PAL rods were observed from the SEM image of RPAL, and the rods are present as bundles or aggregates. The particles ascribed to associated minerals are present around the rods, and tightly “hug” together with PAL rods to form bulk aggregates. After reaction for 2 h, the number of rods gradually decreased and the length of rods becomes short, and the bulk aggregates of associated minerals transformed as many small particles with well dispersed distribution (Fig. 1b). After extending the reaction time to 4 h, well-dispersed spherical particles, instead of PAL rods, were observed (Fig. 1c). We further prolong the reaction time to 12 h and 24 h, the uniformly dispersed spheres stack together to form much gap and pores among spheres (Fig. 1e,f). Interestingly, the formation of the spheres is accompanied with disappearance of the PAL rods as well as associated minerals, which confirm that the spheres are derived from the transformation of PAL and associated minerals during hydrothermal reaction.

**Figure 2:**
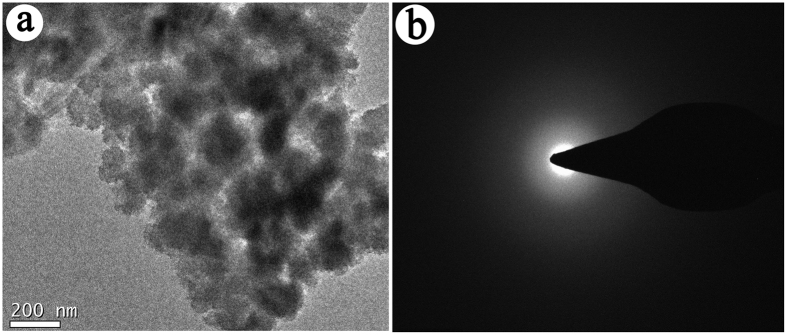
(**a**) TEM image of SiMg-21-12 adsorbent, and (**b**) the respective SAED pattern.

In order to study the effect of Si/Mg ratio on the morphology of the silicate adsorbent, SEM analyses were conducted to the adsorbents prepared at different Si/Mg ratios (reaction time was fixed at 12 h). As shown in Fig. S2 (Supplementary Information), no obvious PAL nanorod was observed at Si/Mg ratio of 3:1 (Fig. S2b, Supplementary Information), and the bulk aggregates of PAL and associated minerals were transformed as well-dispersed small particles (Fig. S2b). This reveals that the crystal structure of PAL rod was broken and transformed as new silicates under alkali condition (the pH value is about 11.9)^40^. For the Si/Mg ratio of 2:1, the PAL nanorods disappeared and spherical particles were observed (Fig. S2c, Supplementary Information), indicating the Si/Mg ratio of 2:1 is suitable for the formation of spherical morphology. The PAL rods were observed (Fig. S2d–f, Supplementary Information) after decreasing Si/Mg ratio to 1:1, 1:2 and 1:3, respectively, indicating that excess of Mg^2+^ ions may decrease the pH value of the reaction medium and thus restrict the structure evolution of PAL crystal. It was found that the pH values of the reaction medium for Si/Mg ratio at 3:1 and Si/Mg ratio at 2:1 are 12.6 and 11.9, respectively. At high pH value of 12.6, the PAL was rapidly evolved and crystallized to form bulk silicate aggregates (Fig. S2b, Supplementary Information). At the moderate pH value of 11.9, the dissolution of PAL and associated minerals and the formation of magnesium silicates simultaneously occurred, which is favorable to the uniform growth of the Mg-silicate to form spherical particles^38^,^39^. After hydrothermal reaction, the pH value of Si/Mg ratio at 2:1 was tested to be 11.8 (11.9 before reaction), indicating that constant alkaline condition is important for the formation of the spherical particles. The TEM image of SiMg-21–12 confirms the porous structure of the adsorbent. From Fig. 2a, pores mainly derived from the gap among the stacked particles were observed, but not well ordered. No diffraction rings were observed in the SAED pattern (Fig. 2b), indicating that the crystals of PAL and associated minerals were transformed as amorphous silicate adsorbent.

### XRD analysis

**Figure 3:**
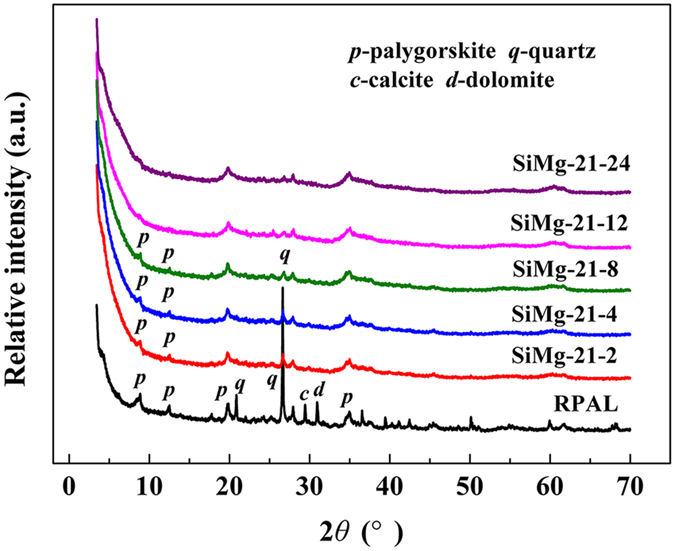
XRD patterns of (**a**) RPAL and the adsorbents (**b**) SiMg-21-2, (**c**) SiMg-21-4, (**d**) SiMg-21-8, (**e**) SiMg-21-12, and (**f**) SiMg-21-24.

The change of crystalline species in PAL clay after hydrothermal reaction was studied by XRD analyses. As shown in Fig. S3 (Supplementary Information), the diffraction peaks at 2*θ* = 8.51° (*d* = 1.1543 nm), 2*θ* = 13.86° (*d* = 0.7096 nm) and 2*θ* = 24.38° (*d* = 0.3651 nm) are ascribed to the (110), (200), and (240) reflection of PAL, respectively. These peaks disappeared after hydrothermal reaction at Si/Mg ratio of 2:1^41^, indicating that crystal of PAL was evolved as new silicate in alkaline medium under hydrothermal condition. Simultaneously, the characteristic diffraction peaks of quartz (2*θ* = 26.87°), calcite (2*θ* = 29.59°) and dolomite (2*θ* = 31.16°) disappeared after hydrothermal reaction, and no new diffraction peaks of crystalline phase were observed. This confirms that both of the PAL crystal and associated minerals (i.e., quartz, calcite and dolomite) transformed into new silicates, which is consistent with the TEM result (Fig. 2). With decreasing the Si/Mg ratio smaller than 1, the characteristic diffraction peaks of PAL, quartz, and dolomite were observed, indicating that there are still crystalline phase of PAL and associated minerals. This means the excess of Mg^2+^ in the reaction system may restrain evolution of PAL and associated minerals. The effect of reaction time on the change of crystalline species after hydrothermal reaction was also studied by XRD analysis, and the results are shown in Fig. 3. The diffraction intensity of (110), (200) and (240) crystalline planes for PAL gradually decreased with prolonging the reaction time. At the reaction time of 12 h, the XRD diffraction peaks of PAL and associated minerals disappeared, and no diffraction peak of Mg_3_Si_2_O_5_(OH)_4_ (JCPDS No. 52-1562) was observed in the XRD pattern (Fig. 3). Small-angle XRD pattern (Fig. S4) shows no peaks for SiMg-21-12 adsorbent. All the above results indicate the amorphous and mesopores features of the hybrid Mg-silicate adsorbent.

### FTIR spectra and chemical composition analyses

**Figure 4:**
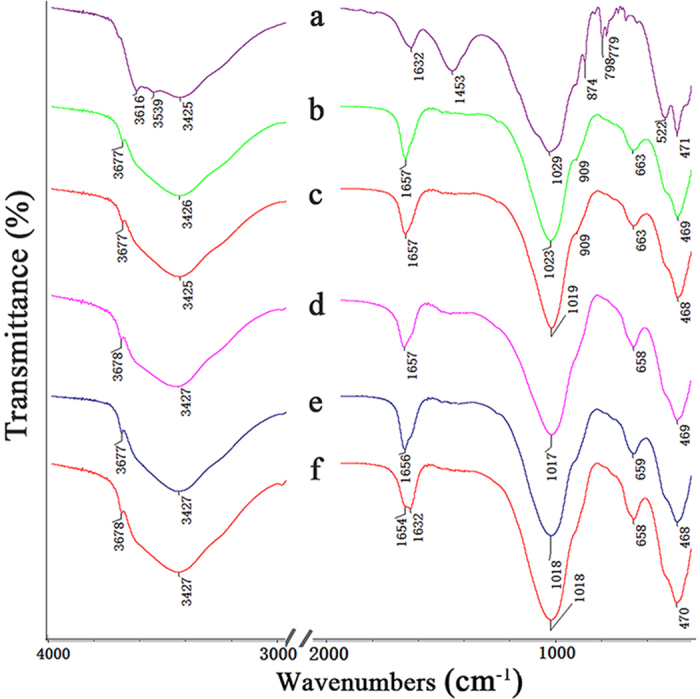
FTIR spectra of (**a**) RPAL and the adsorbents (**b**) SiMg-31-12, (**c**) SiMg-21-12, (**c**) SiMg-11-12, (**d**) SiMg-12-12, and (**e**) SiMg-13-12.

Figure 4 shows the FTIR spectra of RPAL and the hybrid silicate adsorbents prepared at different Si/Mg ratios. The characteristic bands of PAL at 3616 and 3539 cm^−1^ (H-O stretching vibration of Al-OH-Al and Al-OH-Fe), 3425 cm^−1^ (H-O stretching vibration of the water molecules coordinated to Mg cations and the zeolitic water), 1030 cm^−1^ (stretching vibration Si-O-Si in tetrahedron), 909 cm^−1^ (asymmetric stretching vibration of Si-O-Mg), 874 and 663 cm^−1^ (Mg–OH deformation bands), and 1632 cm^−1^ (bending vibration of H-O-H in zeolitic water, coordination and bonding water molecules) were observed (Fig. 4a)^42^,^43^. Also, the SiO_4_ bands of quartz at 798 and 779 cm^−1^, the characteristic bands of dolomite at 1453 cm^−1^ were observed in the spectrum of RPAL (Fig. 4a), which indicate the associated minerals (i.e., quartz, dolomite) exist in RPAL. After hydrothermal reaction, the bands of PAL at 3616 and 3539 cm^–1^, and the band at 874 cm^−1^ disappeared, and the band at 1632 cm^−1^ also shifts to 1657 cm^−1^ (for SiMg-31-12, Fig. 3b), 1657 cm^−1^ (for SiMg-21-12, Fig. 3c), 1657 cm^−1^ (for SiMg-11-12, Fig. 4d), 1656 cm^−1^ (for SiMg-12-12, Fig. 4e) and 1654 cm^−1^ (for SiMg-13-12, Fig. 3f), which indicate the ribbon-layer structure of PAL was damaged during reaction^44^. Simultaneously, the bands of carbonate (at 1453 cm^−1^) and quartz (at 779 and 798 cm^−1^) disappeared after hydrothermal reaction when Si/Mg ratio is 3:1 and 2:1 (Fig. 4b,c), indicating that the associated minerals were removed during reaction. The new band at about 3677 cm^–1^ (the Mg_3_O-H stretching vibration) appeared in the spectra of the adsorbents, whose position is consistent with that of neat magnesium silicate (Fig. S5, Supplementary Information), indicating that Mg-silicate was formed.

X-ray fluorescence (XRF) analysis was used to calculate the ratios of different elements. The Si/Mg molar ratios for the adsorbents are 9.34 (for RPAL), 3.54 (for SiMg-31-12), 2.80 (for SiMg-21-12), 2.06 (for SiMg-11-12), and 1.54 (for SiMg-12-12); the Si/Al ratios are 3.60 (for RPAL), 4.33 (for SiMg-31-12), 5.05 (for SiMg-21-12), 4.81 (for SiMg-11-12), and 4.87 (for SiMg-12-12). The Si/Mg and Si/Al ratios in the optimal adsorbent are determined to be 2.8 and 5.05, respectively.

### BET textural analysis

**Figure 5:**
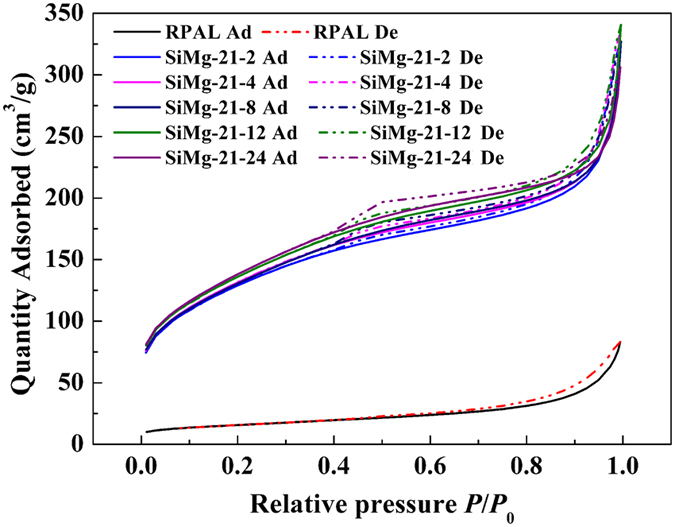
N_2_ adsorption–desorption isotherms of RPAL and the silicate adsorbents.

The textural structure features of RPAL and the hybrid silicate adsorbents were studied using the N_2_ adsorption–desorption isotherms at 77 K. As shown in Fig. 5, the N_2_ quantity adsorbed by each adsorbent gradually increases with increasing relative pressure (*P*/*P*_0_), and the N_2_ adsorption and desorption curves are overlapped in the region of *P*/*P*_0_ ≤ 0.40. The N_2_ quantity adsorbed at *P*/*P*_0_ = 0.40 are 20.05 cm^3^/g (for RPAL), 157.44 cm^3^/g (for SiMg-21-2), 162.01 cm^3^/g (for SiMg-21-4), 162.44 cm^3^/g (for SiMg-21-8), 169.16 cm^3^/g (for SiMg-21-12) and 172.38 cm^3^/g (for SiMg-21-24), respectively. The overlap of adsorption and desorption curves in the region of *P/P*_0_ < 0.4 confirms the monolayer adsorption of N_2_, which is the characteristic of micropores^45^. When *P*/*P*_0_ > 0.4, the N_2_ quantity adsorbed sharply increased due to the capillary condensation effect of N_2_ in the pores, and the typical H3-type hysteresis was observed. These results confirm the presence of mesopores (and/or macropores) in the adsorbents^46^, and the pores in the adsorbents are narrow slit-like pores formed from the aggregates of particles^47^. According to IUPAC classification, the N_2_ adsorption-desorption isotherm of RPAL was classified as Type-IIB with H3-type hysteresis^48^,^49^. This type of isotherm has also been observed in other clay-based materials with a house-of-cards packing and macroporous aggregate^50^. The silicate adsorbents exhibited type-IV adsorption isotherm, with relatively broader H3-type hysteresis, indicating that there are more mesopores in the adsorbent than in RPAL.

**Figure 6:**
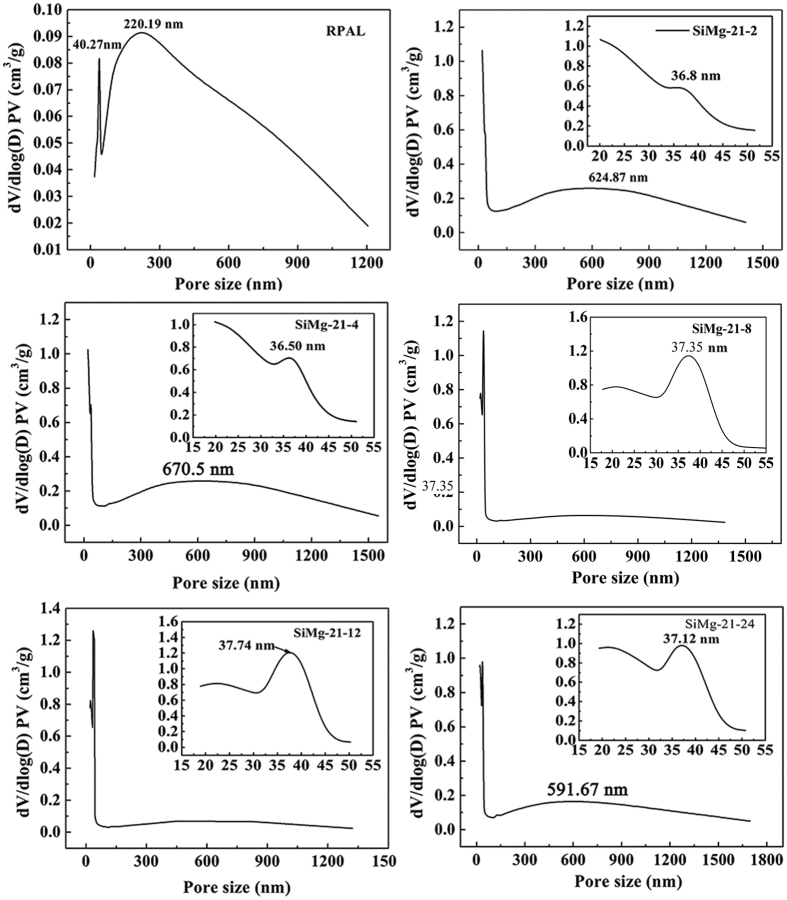
Pore size distribution curves of RPAL and the hybrid silicate adsorbents.

The pore size distribution (PSD) was calculated by BJH (Barret–Joyner–Halenda) method, and the results are shown in Fig. 6. Two peaks were observed in the PSD curve of RPAL: the peak centered at 40.27 nm, attributed to the close-packed particles of the PAL and the associated minerals; the other peak centered at 220.19 nm, attributed to aggregates of bulk particles^51^. The peak centered at 37.74 nm for SiMg-21-12 adsorbent confirms the presence of mesopores, which exhibit high specific surface area of 481.76 m^2^/g (only 54.67 m^2^/g for RPAL) (Table S1, Supplementary Information). As shown in Table S1, tiny amount of micropores (<2 nm) are present in RPAL and the silicate adsorbent. The microporosity can be calculated using Micro% = (*S*_micro_/*S*_total_) × 100%, so the microporosity of RPAL and SiMg-21-12 adsorbent are calculated to be 12.8% and 3.3%, respectively.

### Zeta potential analysis

The inert Si-O-Si (or Al, Fe) bonds in PAL will be broken to form more -Si-O^−^ or -M-O^−^ groups under alkaline condition after hydrothermal reaction, which may affect the distribution of the surface charges^40^. As shown in Fig. S6 (Supplementary Information), the negative Zeta potential of RPAL is −18.6 mV, but it increases to −44.9 mV for SiMg-31-12 and −43.3 mV for SiMg-21-12. The increase of negative surface charges is favorable to intensify the electrostatic attraction between the adsorbent and cationic dyes, which is beneficial to improve the adsorption capacity.

### Adsorption capacities and removal efficiency

**Figure 7:**
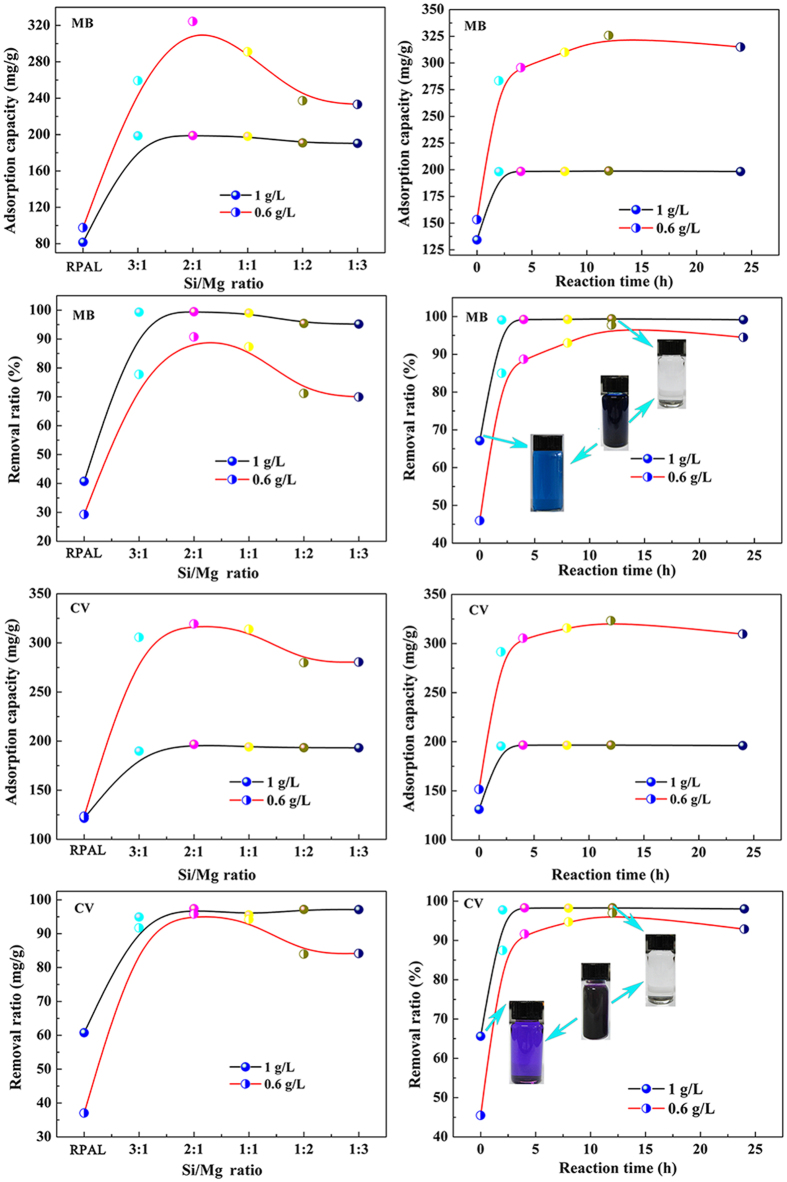
Adsorption capacities and removal efficiency of the hybrid silicate adsorbents prepared at different Si/Mg ratio and reaction time for MB and CV dyes. The initial concentration of dye solution is 200 mg/L.

Figure 7 shows the adsorption capacities and removal efficiency of the silicate adsorbents for MB and CV at the adsorbent dosage of 0.6 g/L and 1 g/L. For both of the MB and CV, the adsorption capacities initially increased with decreasing Si/Mg ratio, reached the maximum at Si/Mg ratio of 2:1, and then decreased. The adsorption capacities of the adsorbents with different Si/Mg ratios follow the sequence of SiMg-21-12 > SiMg-11-12 > SiMg-31-12 > SiMg-12-12 > SiMg-13-12 > RPAL. The SiMg-21-12 adsorbent shows the best adsorption capacities of 324.5 mg/g and 319.3 mg/g for MB and CV, respectively, which is obviously higher than 97.63 mg/g (for MB) and 113.52 mg/g (for CV) of RPAL. Reaction time has relatively smaller influence on adsorption properties. At the reaction time of 2 h, the adsorption capacities of the resultant adsorbent increased to 283.4 mg/g for MB and 292 mg/g for CV. Above 2 h, the increasing trend of adsorption capacity becomes flat with increasing the reaction time. The optimal adsorbent was obtained at the reaction time of 12 h.

Recently, there has been a growing interest in the development of adsorbents that can remove most of pollutants from contaminated water. Figure 7 shows that the adsorbents have excellent removal efficiency for both of the MB and CV. With 99.4% of MB and 99.6% of CV were removed from 200 mg/L of dye solution using only 1 g/L of the adsorbent. In contrast, the removal efficiency of RPAL for MB and CV are 44.1% and 64.3%, respectively under the same condition. The digital photographs of dye solution before and after adsorption also show that the dye solution becomes colorless after treating with SiMg-21-12 adsorbent (the inset in Fig. 7). The relatively stronger adsorption capacity of the silicate adsorbent for dyes than RPAL makes it able to reduce the residue of dye in water to a negligible level, and so could be potentially used for the deep purification of dye wastewater.

### Adsorption isotherms

**Figure 8:**
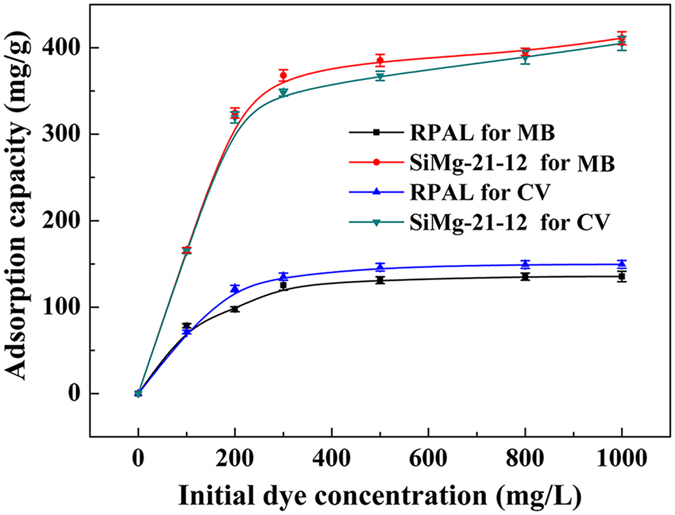
Effect of initial concentrations on the adsorption capacity of RPAL and the optimal silicate adsorbent for MB and CV.

Figure 8 shows variation of adsorption capacities of the silicate adsorbent with increasing the initial dye concentrations. It is obvious that increasing initial dye concentrations facilitates to enhance the saturation adsorption capacities. Since the concentration gradient as the driving force for adsorption at the solid–liquid interface increased with increasing the initial concentration. To explore the adsorption behavior and mechanism, the experimental data were fitted with two typical theoretical models: Langmuir isotherm model (Eq. S1)^52^ and Freundlich isotherm model (Eq. S2)^53^. The corresponding *C*_e_/*q*_e_ *vs C*_e_ curves are shown in Figs S7 and S8 (Supplementary Information), and the adsorption isotherm constants and linear correlation coefficients were calculated from the linear fitting with Langmuir and Freundlich models (Table S2, Supplementary Information). Comparatively, the *C*_e_/*q*_e_ *vs C*_e_ curves fitted with Langmuir model exhibit better linear relation coefficient (*R*^2^ > 0.998) than Freundlich model (Figs S7 and S8); and the adsorption capacities (*q*_e_) determined by experiment are almost equal to the value calculated theoretically (Table S2, Supplementary Information), indicating that the adsorption of the adsorbents for MB and CV dyes follow Langmuir isotherm model well, and the adsorption of MB and CV onto the adsorbent is mainly involved with monolayer coverage^54^.

The monolayer adsorption capacity (*q*_m_) could be calculated from Langmuir isotherm model (Table S2, Supplementary Information), and another adsorption isotherm parameter (*R*_L_) was calculated from Equation S3 55. Herein, the *R*_L_ values of each adsorbent for adsorption of MB and CV are in the range of 0–1, which confirm the adsorption of MB and CV dyes onto the silicate adsorbent is favorable^56^. *R*_L_ value is almost close to zero, indicating the adsorption process was almost invertible, and therefore, strong interactions are present between dyes and the SiMg-21-12 adsorbent.

The maximum adsorption capacities of MB and CV on RPAL are only 143.22 mg/g and 158.76 mg/g, respectively, but which sharply increased to 407.95 mg/g (for MB) and 397.22 mg/g (for CV) after the forming of hybrid silicate adsorbent. Comparatively, the silicate adsorbent was superior to the adsorbents reported previously (Table 1), and could be used as a potential candidate for removal of MB and CV from wastewater.

### Adsorption kinetics

**Figure 9:**
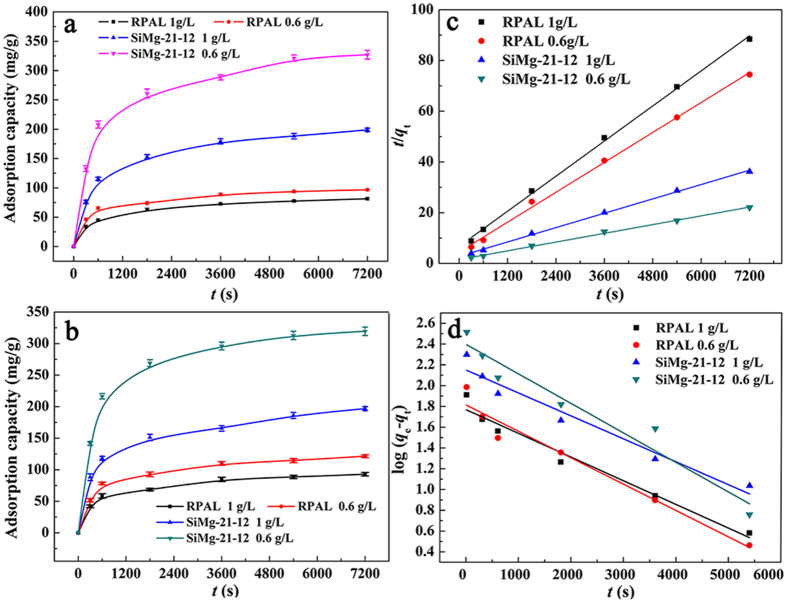
Adsorption kinetic curves of the SiMg-21-12 adsorbent for (**a**) MB and (**b**) CV; and the fitting curves with (**c**) pseudo-second-order model and (**d**) pseudo-first-order model for the adsorption of MB.

As shown in Fig. 9, the adsorption amount of the adsorbents for MB and CV rapidly increased with prolonging the contact time, and reached the adsorption equilibrium within 30 min. In order to get insight into adsorption mechanism (i.e., mass transfer and chemical reaction), the pseudo-first-order (Eq. S4) and pseudo-second-order (Eq. S5) kinetic equations were used to fit the experiment data^57^. *k*_1_ and *k*_2_ values were calculated by the slope and intercept of log(*q*_e_−*q*_t_) *vs t* and *t*/*q*_t_ *vs t* lines, respectively (Table S3, Supplementary Information). The initial adsorption rate constant *k*_2i_ (mg/g/s) is equal to *k*_2_*q*_e_^2^ (Table S3, Supplementary Information). The theoretical *q*_e_ values obtained by fitting with pseudo-first-order kinetic model are far away from the experimental values, and the linear correlation coefficient is smaller than 0.9704 (Fig. 9d and Fig. S9, Supplementary Information). Whereas the theoretical *q*_e_ values obtained by fitting with pseudo-second-order kinetic model would be more close to the experimental values, and the correlation coefficient *R*^*2*^ is larger than 0.9951 (Fig. 9c and Fig. S10, Supplementary Information). This indicates that the adsorption process of MB and CV dyes onto the adsorbent follow pseudo-second-order model very well, and the chemo-adsorption between –Si(Al)-O^–^ groups and dyes mainly contribute to the adsorption. From Table S3 (Supplementary Information), it can be concluded that the initial adsorption rate of the SiMg-21-12 adsorbent for both of the MB and CV is faster than that of RPAL, with the order of SiMg-21-12 (0.6 g/L) > SiMg-21-12 (1 g/L) > RPAL (0.6 mg/L) > RPAL (1 g/L).

Generally, the dye molecules may transfer from bulk solution to the adsorbent by a diffusion process. The possibility of the intra-particle diffusion could be evaluated by using the intra-particle diffusion model (Eq. 1) proposed by Weber and Morris^58^:


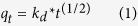


where *q*_t_ is the amount of dyes adsorbed on the adsorbent at time *t, k*_d_ is the intra-particle diffusion rate constant. The plots of *q*_t_ versus *t*^(1/2)^ based on the experimental data are shown in Figs S11 and S12 (Supplementary Information). As can be seen, two steps were observed in the curves: the initial step is due to the external surface adsorption resulting from the boundary layer diffusion effects; the second steps with less slope are the gradual adsorption resulting from intra-particle diffusion^59^. This indicates the diffusion of dyes onto the surface of adsorbent, resulting from the pore adsorption and electrostatic attraction, is relatively faster; while the intra-particle diffusion is the main rate-controlling step.

### Effect of different factors on adsorption and proposed adsorption mechanism

There are four kinds of interactions contribute to the adsorption process: pore adsorption, electrostatic attraction, ion-exchange, and chemical association between surface groups and adsorbates. In order to get insight into the adsorption of the silicate adsorbent for dyes, the FTIR spectra, N_2_ adsorption-desorption, TGA curves, Zeta potential of the MB(or CV)-loaded SiMg-21-12 adsorbent were studied. From the FTIR spectrogram (Fig. S13, Supplementary Information), the characteristic bands of MB at 1600 cm^−1^ (vibration of the aromatic ring), 1492 cm^−1^ and 1399 cm^−1^ (C-N stretching vibration), and 1256 cm^−1^ (C-H in plane and out of plane bending vibration) are slightly shift to 1606 cm^−1^, 1489 cm^−1^ and 1394 cm^−1^, and 1240 cm^−1^, respectively after the adsorption (Fig. S13a,b). This confirms that the hydrogen-bonding or electrostatic interaction are present between MB and the adsorbent^60^. Similarly, the absorbance bands of CV (the aromatic skeletal vibration bands at around 1300–1600 cm^−1^; the C = N stretching at 1587 cm^−1^; the absorbance band of C-N bonds at 1479 cm^−1^) are also shifted after the adsorption (Fig. S13c,d), which indicate the strong interaction also exists between CV and the adsorbent^60^.

After the adsorption of dyes, the N_2_ adsorption-desorption isotherms can be classified as Type-IV with H3-type hysteresis according to IUPAC classification (Fig. S14, Supplementary Information)^48^. The PSD peak of SiMg-21-12 centered at 37.74 nm greatly weakened after the dye adsorption, resulting from the pore was blocked by dye molecules (Fig. S15, Supplementary Information), indicating that the pore-adsorption contributes to the adsorption of cationic dyes. The broad peak centered at about 511 nm in PSD curve appeared after adsorption of dyes, which is caused by the re-aggregation of microsphere induced by dye adsorption, as confirmed by SEM image of the MB-loaded adsorbent (Fig. S16, Supplementary Information). As discussed above, there are more –Si(or Mg, Al)-O^−^ groups in the SiMg-21-12 adsorbent than in RPAL, which may bind or coordinate with dye molecules to greatly enhance the adsorption capacity^61^. The adsorbent with high negative Zeta potential value (−43.3 mV) is favorable to enhance the electrostatic attraction between the adsorbent and cationic dye molecules and thus improve the adsorption capacity. Figure S17 (Supplementary Information) shows the TGA curves for SiMg-21-12 adsorbent and the MB (or CV)-loaded adsorbent. The total weight loss of MB(or CV)-loaded adsorbent is larger than that of SiMg-21-12 due to decomposition of the dyes loaded on the adsorbent. At the temperature range below 542.2 °C, the weight loss of MB (or CV)-loaded adsorbent is lower than that of SiMg-21-12, indicating the stronger interaction of the adsorbent with dye molecules than water molecules, just like the “host-guest” interaction in Maya blue pigment^62^.

**Figure 10:**
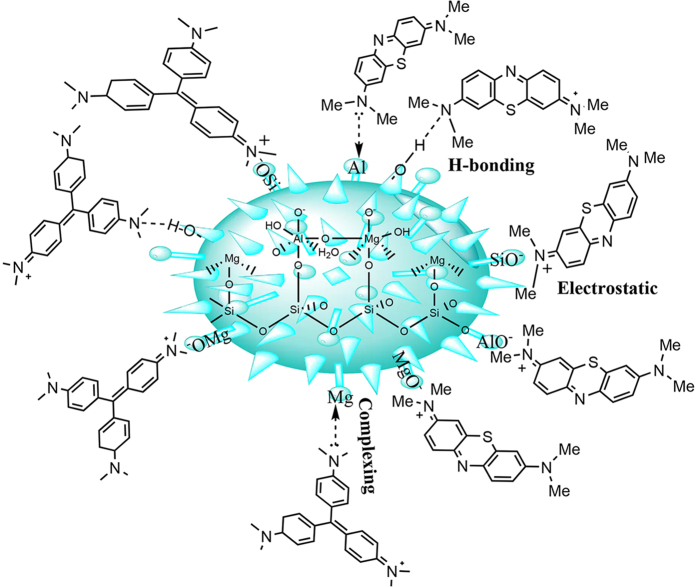
Proposed adsorption mechanism of the adsorbent for MB and CV.

In addition, the cation exchange capacity (CEC) of SiMg-21-12 (42 meq/100 g) is higher than that of RPAL (13.5 meq/100 g), which is consistent with the change tendency of adsorption capacity, indicating the ion exchange also contributes to the adsorption. In summary, the chemical association between the –Si(or Mg, Al)–O^–^ groups and the dyes, electrostatic interaction, ion-exchange, and the pore adsorption all contribute to the adsorption. Among them, the chemical association and electrostatic attraction are mainly responsible for the high adsorption capacity of the adsorbent (Fig. 10).

### Regeneration and reuse of adsorbent

Previous works confirmed that the adsorbents could be regenerated and reused by removal of the adsorbed dyes or heavy metals^63^,^64^. However, due to the strong interaction between dye and hybrid silicate adsorbent, it is difficult to desorb dye by conventional elution method. As reported previously, heat treatment is an effective method to regenerate the spent adsorbent containing organic matters^65^. As to the dye-loaded adsorbent, heat treatment is also feasible to regenerate the spent adsorbent. So, we calcined the dye-loaded adsorbent at 300 °C for 1 h to produce new adsorbent. As shown in Fig. S18 (Supplementary Information), the regenerated adsorbents (denoted as MB-Re1 and CV-Re1) can adsorb 68 mg/g (for MB-Re1) and 73 mg/g (for CV-Re1) of Cu(II); 189.3 mg/g (for MB-Re1) and 182.5 mg/g (for CV-Re1) of MB; 174.3 mg/g (for MB-Re1) and 166.1 mg/g (for CV-Re1) of aureomycin (AMC) from the solution with initial concentration of 200 mg/L. The adsorbent after adsorption of dye or AMC can be further regenerated for several cycles. It can be seen from Fig. S19 (Supplementary Information) that the adsorption capacities of the MB-loaded adsorbent after regenerated for 5 times can still reach 172.1 mg/g (for MB) and 164.7 mg/g (for AMC), respectively.

## Conclusions

As inspired by the sustainable idea of “from nature, for nature”, the environmentally benign mesoporous silicate adsorbent with excellent adsorption capability for cationic dyes was successfully fabricated by one-step transformation of low-grade PAL and the useless associated minerals (i.e., clinochlore, quartz, calcite and dolomite). The newly formed amorphous silicate has high specific surface area of 489.81 m^2^/g and negative surface potential value of −43.3 mV, which is much better than that of raw PAL. The moderate pH value of 11.9 is required to form the mesoporous hybrid silicate adsorbent. The maximum adsorption capacities of the adsorbent for MB and CV reached 407.95 mg/g and 397.22 mg/g, respectively, and the Si/Mg ratio of 2:1 and reaction time of 12 h are the optimal condition to prepare the best adsorbent. High removal efficiency of 99.8% for MB (only 53% for raw PAL) and 99.7% for CV (only 43% for raw PAL) were achieved with 200 mg/L of dye solution by using only 1 g/L of the adsorbent. The chemical association, electrostatic, ion-exchange, and pore adsorption contribute to the enhanced adsorption performance. As a whole, the silicate adsorbents are derived from low-cost PAL minerals and composed of the environmentally benign elements in nature (i.e., O, Si, Al, Fe, Na, Mg), which make it potential to be used as eco-friendly adsorbent for remediation of water environment.

## Experimental

### Materials

Natural red palygorskite (PAL) clay was taken from Huining county, Gansu province of China. Its chemical composition is: Al_2_O_3_, 17.52%; Na_2_O, 3.31%; MgO, 5.96%; CaO, 4.36%; SiO_2_, 67.78%; K_2_O, 3.62%; Fe_2_O_3_, 6.46%. The content of PAL in the mineral is determined to be about 41.3%. Before use, PAL clay was smashed as powder and passed through a 200-mesh sieve. Methylene Blue (MB, A.R. grade) and Crystal violet (CV, A.R. grade) was purchased from Sinopharm Chemical Reagent Co., Ltd (Shanghai, China). Magnesium sulfate heptahydrate (MgSO_4_∙7H_2_O, A.R. grade), sodium metasilicate nonahydrate (NaSiO_3_∙9H_2_O, A.R. grade) were purchased from Aladdin Reagent Inc. (Shanghai, China). All other reagents are of analytical grade and all solution was prepared using deionized water.

### Synthesis of mesoporous hybrid silicate absorbent

PAL powder (1 g) was uniformly dispersed in 25 mL of aqueous solution of Na_2_SiO_3_·9H_2_O (7.8 g) to form a homogeneous suspension, and then 20 mL of aqueous solution of MgSO_4_·7H_2_O (3.38 g) was added dropwise to the resultant suspension under continuous stirring. Finally, the mixture was transferred into an autoclave with Teflon inner, sealed and reacted at 180 °C for different time (2 h, 4 h, 8 h, 12 h, and 24 h). A series of samples with different Si/Mg dosage ratio (3:1, 2:1, 1:1, 1:2, and 1:3) were also prepared at the reaction time of 12 h. The reaction product was fully washed with deionized water to remove the residual free ions, and separated by centrifuge at 5000 rpm, and then dried under vacuum at 60 °C, smashed and passed through a 200-mesh sieve. The natural low-grade PAL was coded as RPAL, and the adsorbents prepared at different reaction time (Si/Mg ratio is fixed at 2:1) were coded as SiMg-21-2 (reaction time, 2 h), SiMg-21-4 (reaction time, 4 h), SiMg-21-8 (reaction time, 8 h), SiMg-21-12 (reaction time, 12 h), and SiMg-21-24 (reaction time, 24 h); the adsorbents prepared at different Si/Mg ratio (reaction time is fixed at 12 h) were coded as SiMg-31-12 (Si/Mg ratio, 3:1), SiMg-21-12 (Si/Mg ratio, 2:1), SiMg-11-12 (Si/Mg ratio, 1:1), SiMg-12-12 (Si/Mg ratio, 1:2), SiMg-13-12 (Si/Mg ratio, 1:3).

### Adsorption experiments

0.015 g (or 0.025 g) of the silicate adsorbents were fully contacted with 25 mL of MB (or CV) solution in a thermostatic shaker (THZ-98A) at 120 r/min and 30 °C to reach adsorption equilibrium. The solution was rapidly separated from the adsorbent by a 0.22 um filter. The concentration of MB (or CV) solution before and after the adsorption was measured by measuring the absorbance of solution at the maximum wavelength of 664 nm (for MB) and 583 nm (for CV) using a UV-*vis* spectroscopy. The adsorption capacity of the adsorbents for MB (or CV) can be calculated by the following equation (2):


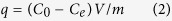


where *q* is the amount of MB (or CV) adsorbed by per unit mass of adsorbents at equilibrium (*q*_e_, mg/g) or time *t (q*_t_, mg/g), *V* is the volume of MB (or CV) solution used (mL), *C*_0_ and *C*_e_ are the initial and final concentration of MB (or CV) solution (mg/L) and *m* is the mass of adsorbent used (mg). The aqueous solution of MB (or CV) with the initial concentration of 200 mg/L was used to evaluate the adsorption capacities, removal efficiency.

The adsorption kinetics was evaluated using the following procedure: 25 mL of the MB (or CV) solution (pH 6.8; initial concentrations, 200 mg/L) was fully contacted with 0.025 g (or 0.015 g) of adsorbent, and then the solution was separated from the adsorbent by filtering through a filter at different intervals (5–240 min). The adsorption amount of the adsorbent for MB (or CV) could be determined by the method described above and calculated with Eq. (2). All experiments were parallel conducted for three times, and the averages are reported in this paper.

### Characterizations

The surface morphology of samples was observed using a field emission scanning electron microscope (FESEM, JSM–6701 F, JEOL, Ltd. Japan) after the samples were fixed on copper stubs and coated with gold film. The XRD patterns were collected using an X’Pert PRO diffractometer equipped with a Cu Kα radiation source (40 kV, 40 mA). TEM was observed using a JEM-1200 EX/S transmission electron microscope (TEM) (JEOL, Tokyo, Japan). FTIR spectra were measured on a Thermo Nicolet NEXUS TM spectrophotometer in the range of 4000–400 cm^–1^ using a KBr platelet. The specific surface area was measured on ASAP 2010 analyzer (Micromeritics, USA) at 77 K by determining the N_2_ adsorption-desorption isotherms. The values of specific surface area (*S*_BET_) were calculated by the BET equation. The total pore volumes (*V*_total_) were obtained from the volume of liquid N_2_ held at the relative pressure *P*/*P*_0_ = 0.95. The micropore volume (*V*_micro_) was estimated by the *t*-plot method. The chemical composition was determined using a Minipal 4 X-ray fluorescence spectrometer (PANalytical, Netherland). Zeta potentials were measured on a Malvern Zetasizer Nano system with irradiation from a 633 nm He-Ne laser (ZEN3600). Before measurement, samples were dispersed in deionized water by a high-speed stirring to form a uniform 0.5% (*w/v*) aqueous dispersion.

## Additional Information

**How to cite this article**: Wang, W. *et al*. All-into-one strategy to synthesize mesoporous hybrid silicate microspheres from naturally rich red palygorskite clay as high-efficient adsorbents. *Sci. Rep.* **6**, 39599; doi: 10.1038/srep39599 (2016).

**Publisher's note:** Springer Nature remains neutral with regard to jurisdictional claims in published maps and institutional affiliations.

## Supplementary Material

Supplementary Information

## Figures and Tables

**Table 1:** Comparison of the adsorption capacities of different adsorbents for MB and CV dyes.

Adsorbents	Adsorbates	*q*_m_ (mg/g)	Ref.
Attapulgite/bentonite (50%)	MB	168.63	66
Titanate nanotubes	MB	133.3	60
Grinding palygorskite	MB	111.78	25
Sepiolite	MB	57.38	67
Powdered activated carbon	MB	91	68
Palygorskite	MB	143.22	This work
SiMg-21-12 silicate adsorbent	MB	407.95	This work
Halloysite nanotubes	CV	113.64	69
Sepiolite	CV	92.58	70
Mansonia wood sawdust	CV	20	71
Rice hull char	CV	123	72
Palygorskite	CV	158.76	This work
SiMg-21-12 silicate adsorbent	CV	397.22	This work
