# Supplementary material for: All-into-one strategy to synthesize mesoporous hybrid silicate microspheres from naturally rich red palygorskite clay as high-efficient adsorbents
Source: Sci Rep. 2016 Dec 21;6:39599. doi: 10.1038/srep39599 (PMC5175148; doi:10.1038/srep39599)
Supplement: Supplementary Information [file srep39599-s1.doc]

**Supplementary Information**

**All-into-one strategy to synthesize mesoporous hybrid silicate microspheres from naturally rich red palygorskite clay as high-efficient adsorbents**

Wenbo Wang,^1,3†^ Guangyan Tian,^1,2†^ Dandan Wang,^1,3^ Zhifang Zhang,^1,2^ Yuru Kang,^1,3^ Li Zong,^1,3^ Aiqin Wang,1,3[[1]](#footnote-2)*

^a^ Key Laboratory of Clay Mineral Applied Research of Gansu Province, Center of Eco-materials and Green Chemistry, Lanzhou Institute of Chemical Physics, Chinese Academy of Sciences, Lanzhou 730000, P.R. China

^b^University of the Chinese Academy of Sciences, Beijing 100049, P.R. China

^c^R&D Center of Xuyi Palygorskite Applied Technology, Lanzhou Institute of Chemical Physics, Chinese Academy of Sciences, Xuyi 211700, P.R. China

**Section I: Adsorption isotherm models and kinetic models**

To further investigate the adsorption process and mechanism, the adsorption data was fitted with two typical theoretical models: Langmuir isotherm model (Eq. S1) and Freundlich model (Eq. S2).

*C*_e_/*q*_e_ = 1/(*q*_m_*b*) + *C*_e_/*q*_m_ (1)

log*q*_e_ = log*K* + (1/*n*)log*C*_e_ (2)

In which, *q*_e_ (mg/g) is the adsorption capacity of the adsorbent for dyes, *C*_e_ (mg/L) is the concentration of dyes in the aqueous solution after adsorption equilibrium. *q*_m_ (mg/g) is the maximum adsorption capacity of the adsorbent that is calculated by the fitting curves with Langmuir model, *b* (L/m) is a constant which is bound up with the adsorption energy. These parameters can be obtained by fitting the experiment data with Langmuir model. *K* is the Freundlich isotherm constant with respect to the binding energy and is defined as the distribution coefficient, and constant *n* usually refers to the adsorption intensity, which can be obtained by fitting the experiment data with Freundlich model.


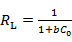
 (3)

where, *b* (L/mol) is the Langmuir adsorption constant and is related to the free energy of adsorption, *C*_0_ (mg/L) is the maximum initial concentration of dye solution, and *R*_L_ is the dimensionless parameter. When *R*_L_>1, the adsorption process is not allowed; when 0<*R*_L_<1, the adsorption process is invertible; when *R*_L_=0, the adsorption process is irreversible.

In order to get insight into the adsorption mechanism (i.e., mass transfer and chemical reaction), the pseudo-first-order (Eq. S4) and pseudo-second-order (Eq. S5) kinetic equations were used to fitting the experiment data.

log(*q*_e_ – *q*_t_) = log*q*_e_ – (*k*_1_/2.303)*t* (4)

*t*/*q*_t_ = 1/*k*_2_*q*_e_^2^ + *t*/*q*_e_ (5)

where *q*_e_ and *q*_t_ are the adsorption capacities of the adsorbents for MB (mg/g) at equilibrium and time *t* (s), respectively. *k*_1_ is the pseudo-first-order rate constant (s^−1^), and *k*_2_ is the pseudo-second-order rate constant (mg/g/s).

**Section II: Supplementary Figures**


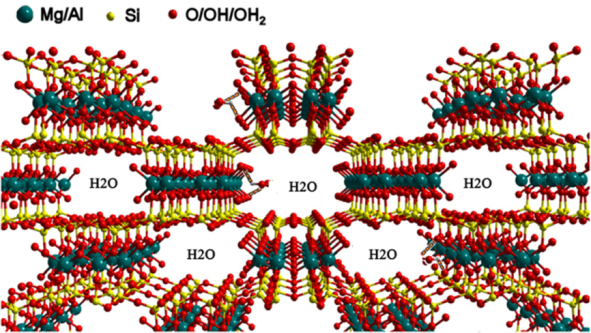


**Fig. S1** Structure of palygorskite with 2:1 ribbon-layer


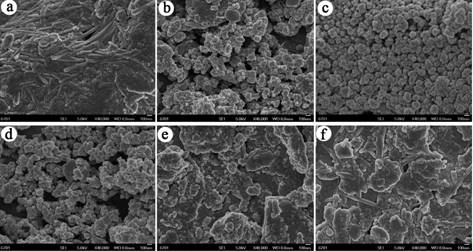


**Fig. S2** The SEM images of (a) RPAL, (b) SiMg-31-12, (c) SiMg-21-12, (d) SiMg-11-12, (e) SiMg-12-12, and (f) SiMg-13-12.


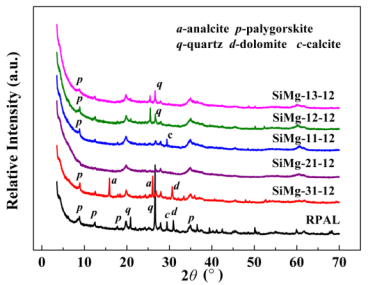


**Fig. S3** XRD patterns of RPAL and the hybrid silicate adsorbents prepared at different Si/Mg ratio


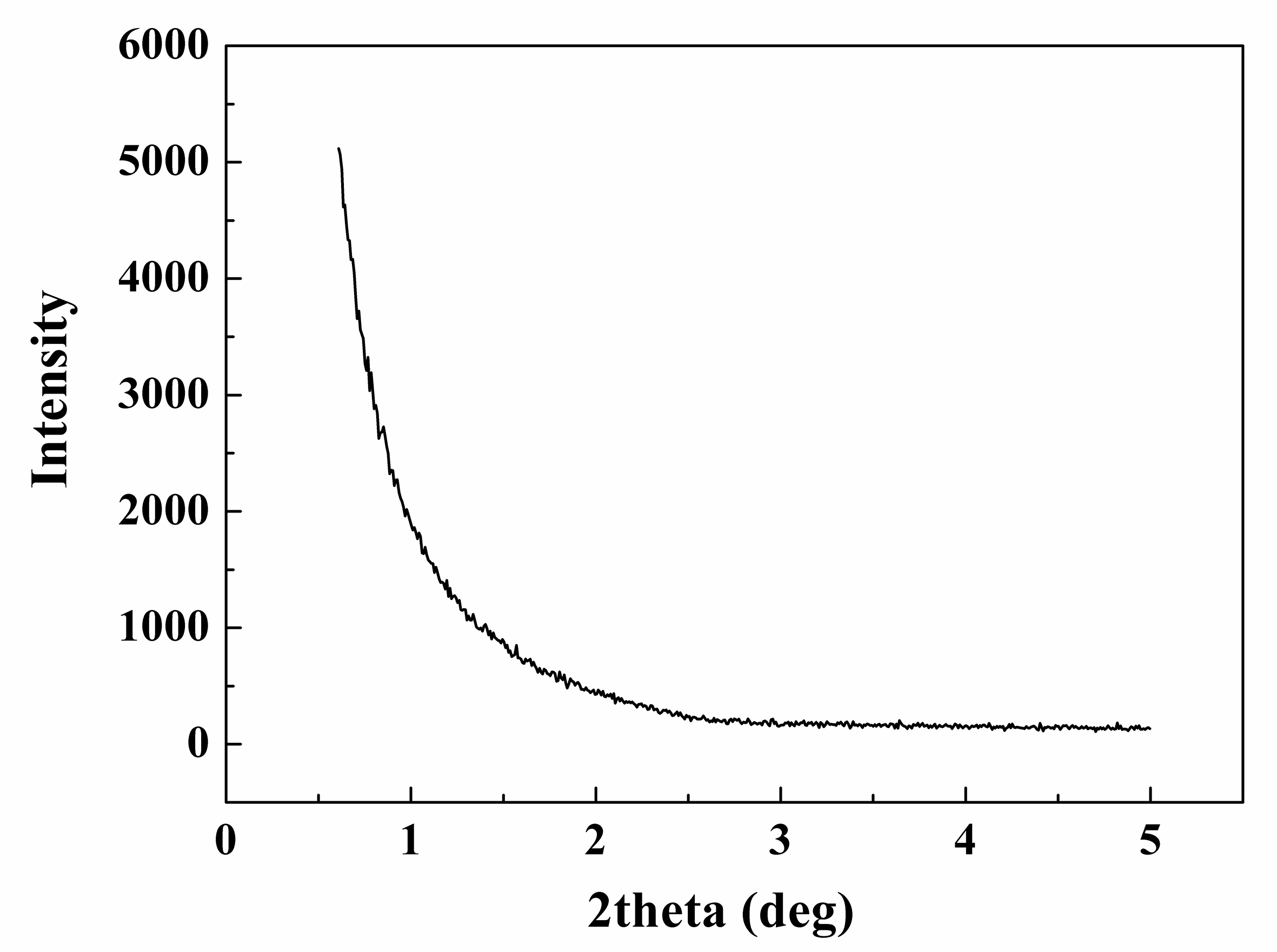


**Fig. S4** Small-angle XRD pattern of the SiMg-21-12 adsorbent


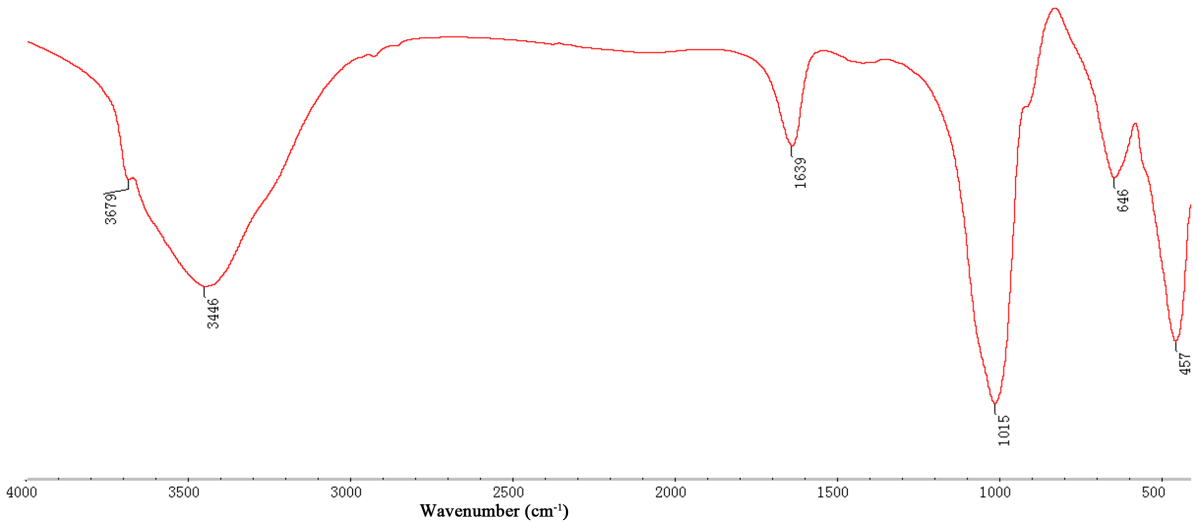


**Fig. S5** FTIR spectrum of neat magnesium silicate


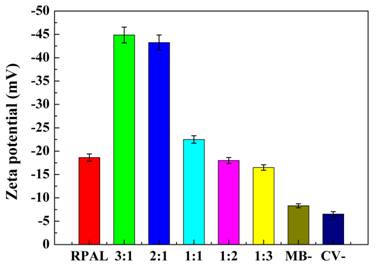


**Fig. S6** Zeta potentials of RPAL, the adsorbents prepared at different Si/Mg ratio and the MB- and CV-loaded SiMg-21-12 adsorbent


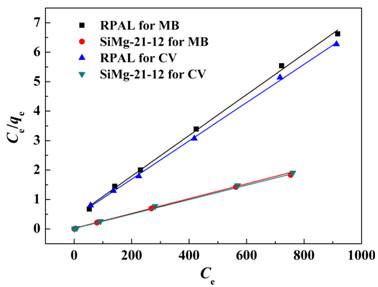


**Fig. S7** The fitting plots of *C*_e_/*q*_e_ versus *C*_e_ with Langmuir isotherm model for the adsorption of MB and CV onto RPAL and the SiMg-21-12 adsorbents.


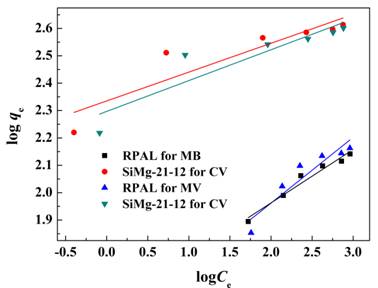


**Fig. S8** The fitting plots of log*q*_e_ versus log*C*_e_ with Freundlich isotherm model for the adsorption of MB and CV onto RPAL and the SiMg-21-12 adsorbents.


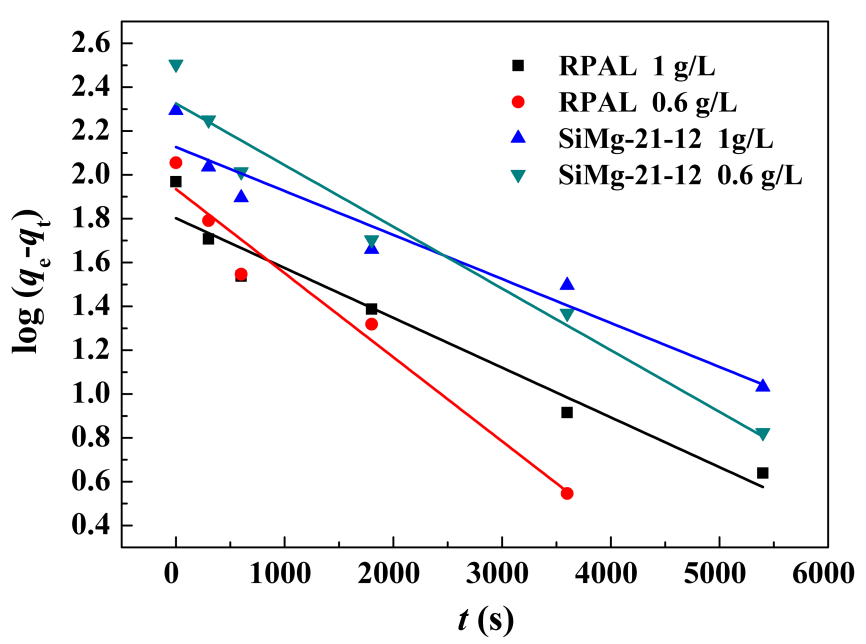


**Fig. S9** The fitting plots of log(*q*_e_*-q*_t_*)* versus *t* by pseudo-first-order kinetic model for the adsorption of CV onto RPAL and the adsorbent. The initial concentration of dye solution is 200 mg/L.


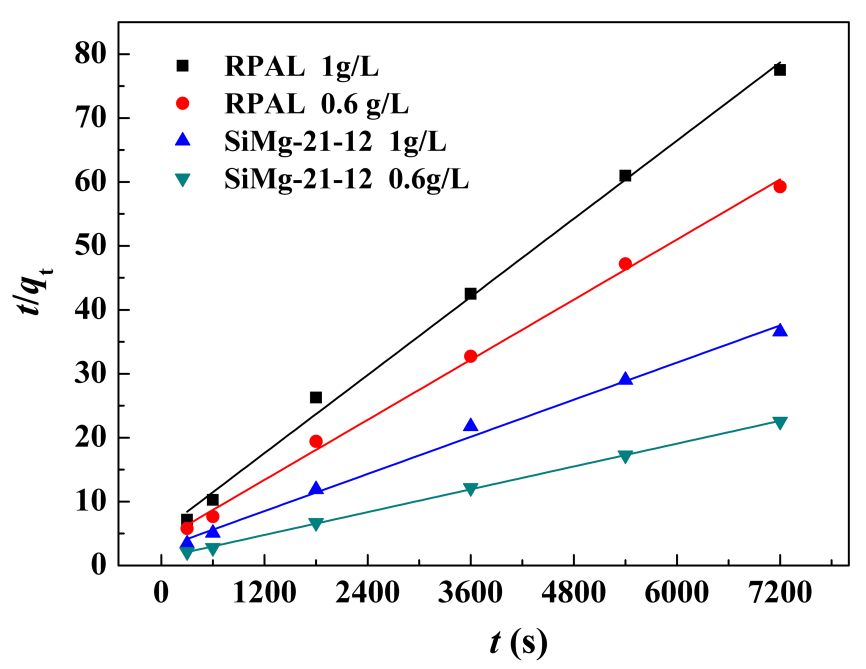


**Fig. S10** The fitting plots of *t*/*q*_t_ versus *t* by pseudo-second-order kinetic model for the adsorption of CV onto RPAL and the SiMg-21-12 adsorbent at different dosages. The initial concentration of dye solution is 200 mg/L.


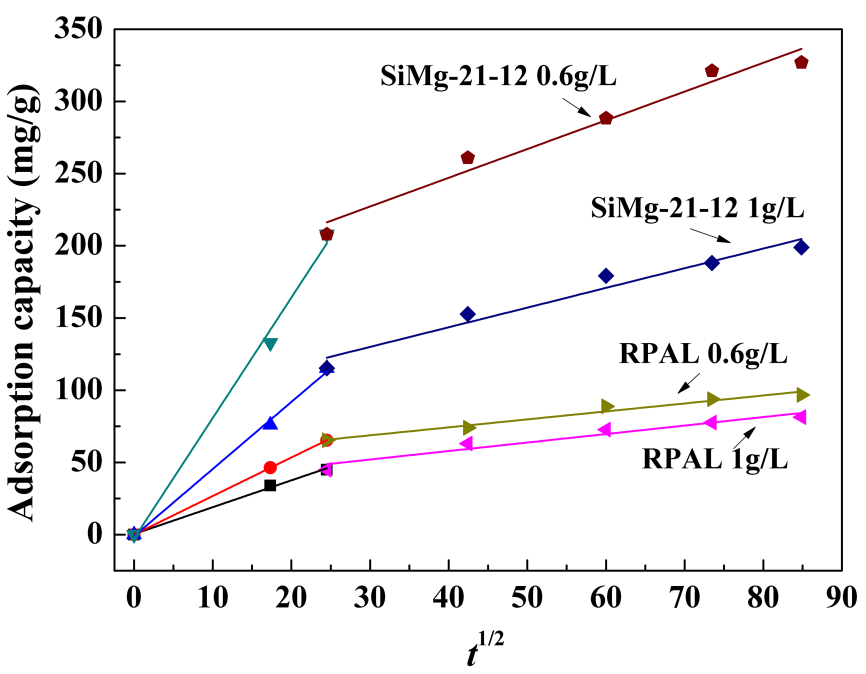


**Fig. S11** Weber–Morris equation plot for the adsorption of MB onto the SiMg-21-12 adsorbent. The initial concentration of dye solution is 200 mg/L.


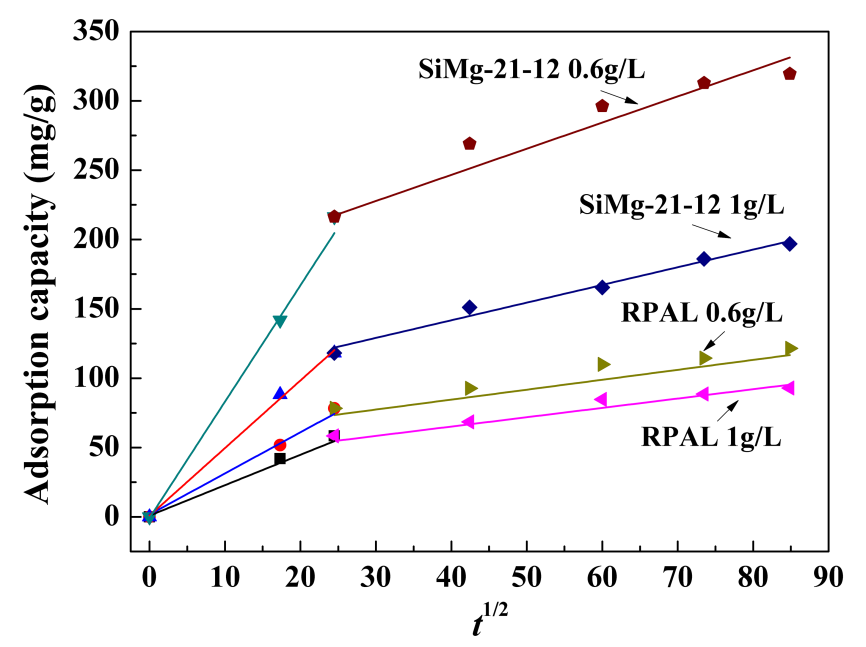


**Fig. S12** Weber–Morris equation plot for the adsorption of CV onto the SiMg-21-12 adsorbent. The initial concentration of dye solution is 200 mg/L.


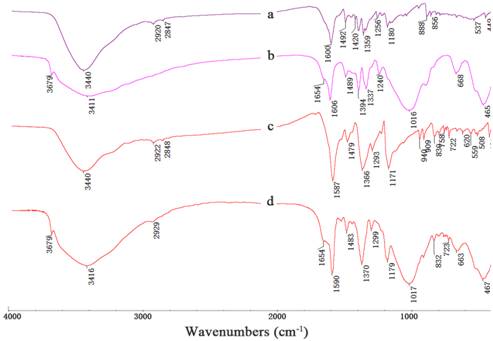


**Fig. S13** FTIR spectra of (a) MB, (b) MB-loaded SiMg-21-12 adsorbent, (c) CV and (d) CV-loaded SiMg-21-12 adsorbent.


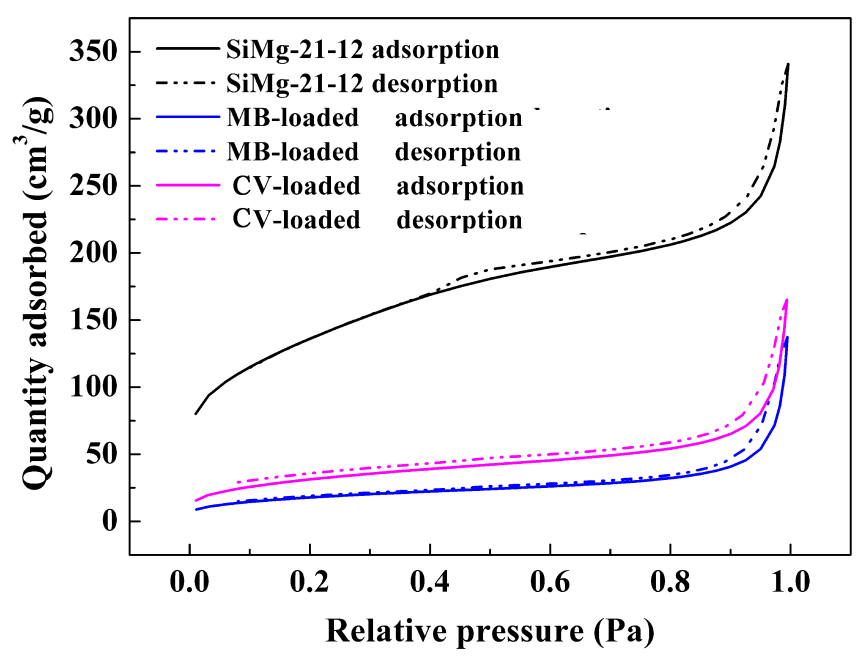


**Fig. S14** N_2_ adsorption–desorption isotherms of the SiMg-21-12 hybrid silicate adsorbent and the MB- and CV-loaded SiMg-21-12 adsorbents

**
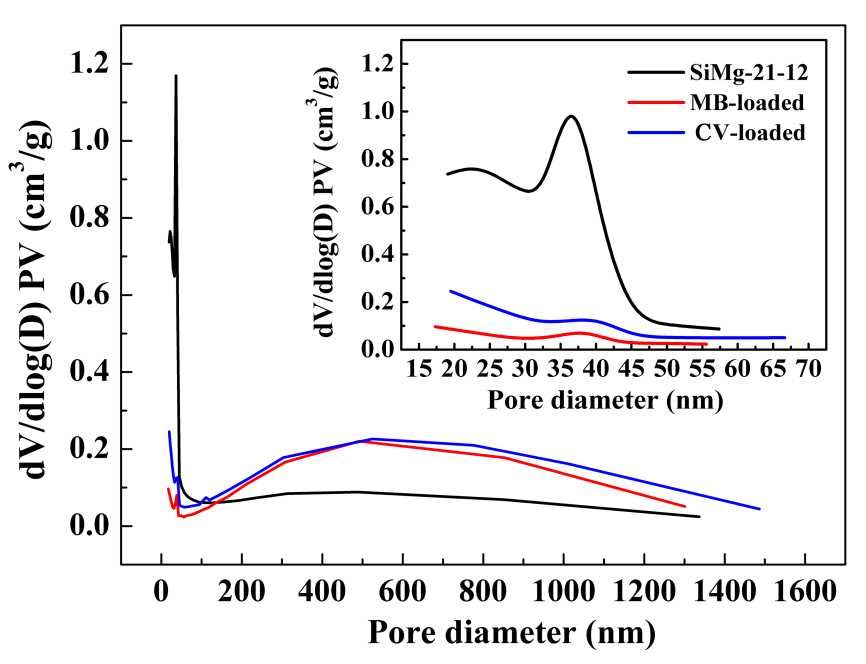
**

**Fig. S15** Pore size distribution curves of the SiMg-21-12 hybrid silicate adsorbent and the MB- and CV-loaded SiMg-21-12 adsorbents

**
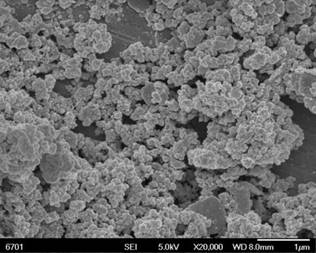
**

**Fig. S16** SEM micrographs of the MB-loaded SiMg-21-12 adsorbent.

**
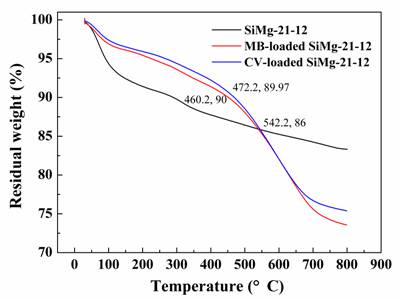
**

**Fig. S17** TGA curves of SiMg-21-12 adsorbent, MB-loaded SiMg-21-12 and CV-loaded SiMg-21-12 adsorbent. The thermogravimetric analysis (TGA) was performed on a Diamond TG-DTA 6300 thermoanalyzer under a N2 atmosphere from 30 to 800°C at a heating rate of 10°C/min.

**
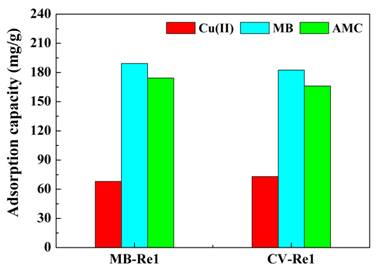
**

**Fig. 18** The adsorption capacities of the regenerated adsorbent for Cu^2+^, MB and aureomycin (AMC).


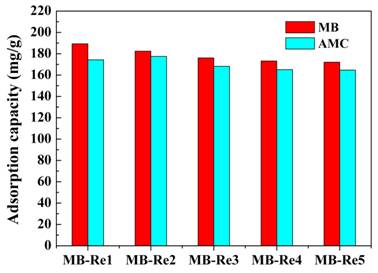


**Fig. 19** The adsorption capacities of the regenerated adsorbent for MB and aureomycin (AMC) after regenerated for 5 times.

**Section III: Supplementary Tables**

**Table S1** Pore structure parameters of RPAL and the hybrid silicate adsorbents

| Samples | *S*_BET_ (m^2^/g) | *S*_micro_ (m^2^/g) | *S*_ext_ (m^2^/g) | *V*_total_ (cm^3^/g) | *V*_micro_(cm^3^/g) | PZ (nm) |
| --- | --- | --- | --- | --- | --- | --- |
| RPAL | 54.67 | 7.00 | 47.67 | 0.1288 | 0.0034 | 9.423 |
| SiMg-21-2 | 452.92 | 19.35 | 433.57 | 0.4989 | 0.0099 | 4.406 |
| SiMg-21-4 | 463.72 | 16.13 | 447.59 | 0.5143 | 0.0075 | 4.436 |
| SiMg-21-8 | 480.81 | 15.96 | 464.85 | 0.5268 | 0.0073 | 4.388 |
| SiMg-21-12 | 481.76 | 15.91 | 465.85 | 0.5270 | 0.0071 | 4.385 |
| SiMg-21-24 | 489.81 | 9.64 | 480.17 | 0.4737 | 0.0035 | 3.868 |
| MB-SiMg-21-12 | 64.60 | 9.55 | 55.05 | 0.2123 | 0.0035 | 13.147 |
| CV-SiMg-21-12 | 112.90 | 16.80 | 96.10 | 0.2554 | 0.0064 | 9.049 |

**Table S2** Adsorption isotherm parameters for adsorption of MB and CV dyes

| Samples | Dyes |  | Langmuir Model | | |  | | |
| --- | --- | --- | --- | --- | --- | --- | --- | --- |
|  |  | R_L_ | *q*_m_ | *b* |  | Freundlich Model | | |
|  |  |  | mg/g | L/mg | *R*^2^ | *K* | *n* | *R*^2^ |
| RPAL | MB | 0.0441 | 143.22 | 0.0217 | 0.9985 | 30.29 | 4.1617 | 0.8532 |
| SiMg-21-12 | MB | 0.0064 | 407.95 | 0.1554 | 0.9980 | 198.04 | 8.8716 | 0.8045 |
| RPAL | CV | 0.0431 | 158.76 | 0.0222 | 0.9989 | 37.37 | 5.1258 | 0.9567 |
| SiMg-21-12 | CV | 0.0093 | 397.22 | 0.1066 | 0.9990 | 216.29 | 9.4888 | 0.8015 |

**Table S3** Adsorption kinetic parameters for adsorption of MB and CV dyes.

| Samples | Dyes | Dosage (g/L) | Pseudo-first-order model | | | | Pseudo-second-order model | | | |
| --- | --- | --- | --- | --- | --- | --- | --- | --- | --- | --- |
|  |  |  | *q_e_,* _exp_ | *q*_e_, _cal_ | *k*_1_ | *R*^2^ | *q*_e_, _cal_ | *k*_2_×10^5^ | *k*_2i_ | *R*^2^ |
|  |  |  | mg/g | mg/g | min^-1^ |  | mg/g | g/(mg∙s) | mg/(g∙s) |  |
| RPAL | MB | 1.0 | 81.41 | 58.36 | 0.0315 | 0.9629 | 86.76 | 1.9704 | 0.1483 | 0.9983 |
| SiMg-21-12 | MB | 1.0 | 198.94 | 140.31 | 0.0304 | 0.9475 | 203.97 | 0.7960 | 0.3577 | 0.9984 |
| RPAL | MB | 0.6 | 96.71 | 64.72 | 0.0350 | 0.9526 | 101.76 | 2.1613 | 0.2238 | 0.9973 |
| SiMg-21-12 | MB | 0.6 | 326.90 | 247.17 | 0.0392 | 0.9375 | 347.93 | 0.5480 | 0.6633 | 0.9976 |
| RPAL | CV | 1.0 | 92.91 | 63.39 | 0.0313 | 0.9615 | 98.19 | 1.9323 | 0.1863 | 0.9953 |
| SiMg-21-12 | CV | 1.0 | 198.8 | 133.81 | 0.0277 | 0.9227 | 201.50 | 0.9098 | 0.3694 | 0.9951 |
| RPAL | CV | 0.6 | 113.51 | 86.01 | 0.0530 | 0.9414 | 127.66 | 1.5334 | 0.2499 | 0.9967 |
| SiMg-21-12 | CV | 0.6 | 318.48 | 211.61 | 0.0388 | 0.9704 | 335.95 | 0.7308 | 0.8248 | 0.9994 |

1. ^*^ Corresponding author, Tel.: +86 931 4968118; fax: +869318277088. Email: [aqwang@licp.cas.cn](mailto:aqwang@licp.cas.cn) (A.Q. Wang); ^†^The authors have contributed equally to the paper. [↑](#footnote-ref-2)
